# Synthesis of a ^13^C-methylene-labeled isoleucine precursor as a useful tool for studying protein side-chain interactions and dynamics

**DOI:** 10.1007/s10858-023-00427-2

**Published:** 2023-10-11

**Authors:** Theresa Höfurthner, Giorgia Toscano, Georg Kontaxis, Andreas Beier, Moriz Mayer, Leonhard Geist, Darryl B. McConnell, Harald Weinstabl, Roman Lichtenecker, Robert Konrat

**Affiliations:** 1 Christian Doppler Laboratory for High-Content Structural Biology and Biotechnology, Department of Structural and Computational Biology, Max Perutz Laboratories, University of Vienna, Campus Vienna Biocenter 5, 1030 Vienna, Austria; 2 Christian Doppler Laboratory for High-Content Structural Biology and Biotechnology, Institute of Organic Chemistry, University of Vienna, Währingerstraße 38, 1090 Vienna, Austria; 3 Vienna Doctoral School in Chemistry (DoSChem), University of Vienna, Währingerstraße 42, 1090 Vienna, Austria; 4 Department of Structural and Computational Biology, Max Perutz Laboratories, University of Vienna, Campus Vienna Biocenter 5, 1030 Vienna, Austria; 5 Boehringer Ingelheim RCV GmbH & Co. KG, Dr. Boehringer Gasse 5-11, 1121 Vienna, Austria

**Keywords:** Precursor labeling, Transversal relaxation studies, CH-π interaction, Ligand binding

## Abstract

In this study, we present the synthesis and incorporation of a metabolic isoleucine precursor compound for selective methylene labeling. The utility of this novel α-ketoacid isotopologue is shown by incorporation into the protein Brd4-BD1, which regulates gene expression by binding to acetylated histones. High quality single quantum ^13^C−^1^ H-HSQC were obtained, as well as triple quantum HTQC spectra, which are superior in terms of significantly increased ^13^C-T_2_ times. Additionally, large chemical shift perturbations upon ligand binding were observed. Our study thus proves the great sensitivity of this precursor as a reporter for side-chain dynamic studies and for investigations of CH-π interactions in protein-ligand complexes.

**Supplementary Information:**

The online version contains supplementary material available at 10.1007/s10858-023-00427-2.

## Introduction

Nuclear Magnetic Resonance (NMR) Spectroscopy has matured into a powerful tool to characterize interactions between biological molecules and their ligands at atomic resolution. Although originally described as a versatile technique to screen for potential protein binders (Gossert and Jahnke 2016; Harner et al. 2017; Luchinat et al. 2020; Gronenborn 2022), more sophisticated detection schemes have now emerged. Ligand-based detection schemes, such as waterLOGSY, Saturation Transfer Difference (STD) spectroscopy and ^15^N–^13^C-filtered ^1^H-1D spectra, can reveal valuable information about site specific interaction patterns and their mechanistic details (Dalvit et al. 2000; Mayer and Meyer 2001). Together with protein NMR-based mapping of ligand binding, this unveils unique information about the characteristics of protein-ligand complexes. The potential of protein NMR for drug discovery and design campaigns was realized by the discovery of Structure-Affinity-Relationship (SAR) by NMR by the Fesik group, in which ^1^H–^15^N-HSQCs on uniformly labeled proteins are recorded in order to investigate ligand binding interactions (Shuker et al. 1996). A particularly powerful application of SAR is Fragment-based drug discovery (FBDD) (Rees et al. 2004). FBDD starts with small fragments (< 300 g/mol) of weak binders (mM-100 µM), which are then structurally optimized throughout the process in order to maximize non-covalent interactions, most importantly the Van der Waals, electrostatic, and hydrogen bond interactions. Despite many past applications, backbone (^1^H–^15^N) detection schemes do not fully harness the potential of NMR spectroscopy in drug design campaigns due to limited experimental sensitivity, spectral overlap, and the ambiguous relationship between ligand binding modes and ^1^H^N^–^15^N chemical shift perturbations (Williamson 2013). These problems can be largely overcome by resorting to ^1^H–^13^C side-chain detection exploiting the exquisite sensitivity and spectral dispersion obtained with Methyl-TROSY approaches and multiple quantum coherence experiments, which allow applications to slowly tumbling, high molecular weight proteins (Tugarinov et al. 2003, 2004). Selective labeling techniques for aliphatic and/or aromatic residues have been described and are by now well established (Lichtenecker et al. 2013a, b). Labeling of the aliphatic amino acids Isoleucine, Valine and Leucine was one of the first methods of selective ^13^C incorporation reported in the literature (Cardillo et al. 1977). These amino acids are highly abundant in the hydrophobic cores of proteins and thus represent valuable targets to introduce ^13^C- and ^2^H-nuclei with the intention to reduce signal overlap, increase signal-to-noise ratio and optimize magnetization transfer pathways. Moreover, metabolic α-ketoacid precursors can be added to the minimal growth media of *E.coli*-based overexpression systems and are effectively metabolized into the corresponding target aliphatic amino acids *in-vivo* within defined metabolic pathways (Gardner and Kay 1997; Goto et al. 1999). So far, ^13^C incorporation in aliphatic residues has mainly focused on methyl groups to benefit from their advantageous relaxation properties due to fast C–C bond rotation and the reduction of signal overlap in the otherwise crowded spectral region of methyl signals. Methyl CH_3_ groups and aromatic CHs are sensitive reporters of the ligand binding mode and changes of side-chain structural dynamics upon ligand binding. A special and particularly interesting case of non-covalent interactions are CH-π interactions, in which the CH group of the aliphatic or aromatic residue acts as the hydrogen-bond donor, whereas the π-system is the hydrogen-bond acceptor (Hunter 2004). We have recently shown that CH-π interactions are determinants for the affinity of a drug molecule to its target protein and can efficiently be probed by selective amino acid labeling (Platzer et al. 2020).

Encouraged by our previously obtained results, we suggest extending the selective labeling methodology to the methylene group (CH_2_) of Isoleucine.

**Scheme 1:**
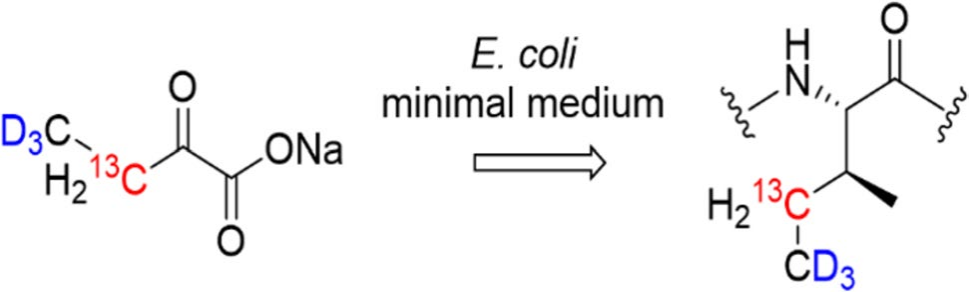
Conversion of [3-^13^C; 4,4,4-^2^H_3_] 2-ketobutyrate in *E. coli* minimal media to Isoleucine

 Herein, we show that γ1-^13^C Isoleucine patterns are effectively induced by the corresponding precursor [3-^13^C, 4,4,4-^2^H_3_] 2-ketobutyrate (Scheme 1) and can be applied as sensitive tools to probe interactions between aryl-ligand CH-π acceptors and protein Isoleucine residue CH-π donors.

**Scheme 2:**
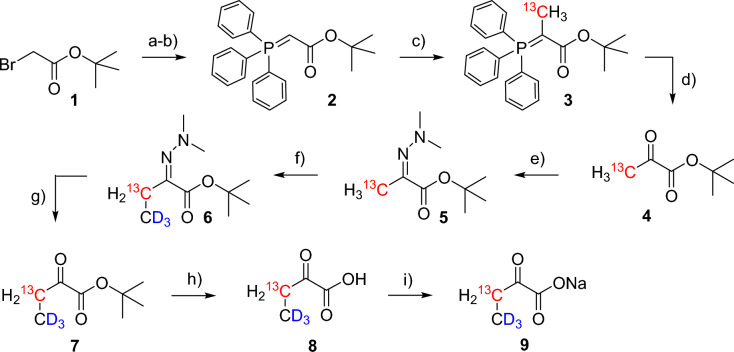
Synthesis of [3-^13^C; 4,4,4-^2^H_3_] sodium α-ketobutyrate **9**. Reagents and conditions: **a** PPh_3_, toluene, rt, overnight; quant.; **b** NaOH_aq_ (1 M), rt, 15 min, quant.; **c** ^13^CH_3_I, DCM, rt, overnight; **d** O_3_, −78 °C, DCM, 5 min, then PPh_3_, 3 h, rt, 70% over two steps; **e** H_2_NNMe_2_, Et_2_O, 18 h, rt, 76%; **f** NaHMDS, CD_3_I, THF, –78 °C, 5 min, 80%; **g** HCl_aq_ (1 M), Et_2_O, 95%; **h** HCl_g_, DCM/Et_2_O 1:1, 18 h, 0 °C-rt, 90%; **i** NaOH_aq_ (1 M), lyophilisation, quant. Experimental details, as well as NMR characterization of products and intermediates are given in the Supplementary Information (SI)

We additionally describe the possibility to use [3-^13^C, 4,4,4-^2^H_3_] 2-ketobutyrate in D_2_O *E.coli* overexpression medium containing *U*-^2^H glucose to increase the deuteration grade in the β and γ2 positions of the Ile-side chains. This additional deuteration attenuates line-broadening due to ^1^H–^1^H dipolar interaction and reduces the number of signals resulting from natural abundance methyl correlations.

## Materials and methods

### Precursor synthesis

Isotopologues of α-ketobutyric acid are common additives in minimal media of bacterial protein overexpression to achieve highly selective Isoleucine labeling. While most of these methods aim for ^13^C methyl groups, we established a novel synthetic route to access an Ile γ_1_-^13^CH_2_/δ-CD_3_ pattern (Scheme 2). Our approach combines a modified literature-known protocol to synthesize 3-^13^C pyruvate (Werkhoven et al. 1999) with an optimized procedure of dimethylhydrazone monomethylation. Starting from *tert*-butyl bromoacetate **1**, the phosphorous ylide **2** was formed and subsequently methylated using ^13^C-iodomethane. Ozonolysis with careful bulb-to-bulb distillation of the product from the viscous reaction residue yielded [3-^13^C] *tert*-butyl pyruvate **4**. This compound was further converted to the dimethylhydrazone **5**, which was then deprotonated at the alpha-position using sodium hexamethyldisilazane (NaHMDS) and subsequently transformed to the hydrazone **6** upon [^2^H_3_] iodomethane addition. Short reaction times in this methylation step effectively minimized the formation of side-products (see SI for experimental details). Alternative protocols using lithium diisopropylamide (LDA) as a base reported in literature could not be reproduced in comparable yields (Hajduk et al. 2000). Final hydrazone hydrolysis and ester cleavage under acidic conditions resulted in the formation of the target compound [3-^13^C; 4,4,4-^2^H_3_] α-ketobutyric acid **8**. Compound **8** can be directly applied to protein overexpression as a free acid or lyophilized to a white powder of the corresponding sodium salt **9**, respectively.

### Ligand synthesis

Ligands **A** (CAS: 1610045-04-9) and **B** (CAS: 1772600-27-7) were synthesized as reported in earlier work (Platzer et al. 2020). The corresponding structural details of these two compounds are shown in the supporting information.

### Expression and purification of Brd4-BD1

Protein overexpression was performed as described previously (Schörghuber et al. 2017). Briefly, recombinant human Brd4-BD1 (bromodomain 1 of Bromodomain containing protein 4) was expressed in *E.coli* BL21 (DE3), which contains an N-terminal TEV-cleavable His6-tag (plasmid was kindly provided by Boehringer Ingelheim GmbH & Co. KG). Uniformly ^15^N and ^13^C-labeled Brd4-BD1 was expressed following the expression protocol for efficient isotopic labeling of recombinant proteins using a fourfold cell concentration in M9 minimal medium, supplemented with 1 g/L ^15^NH_4_Cl, 3 g/L ^13^C_6_-D-glucose. Uniformly ^15^N and selective γ_1_-^13^C Isoleucine labeled Brd4-BD1 was expressed following the same protocol, M9 minimal medium was supplemented with 1 g/L ^15^NH_4_Cl, 3 g/L ^12^C_6_-D-glucose and 130 mg/L [3-^13^C, 4,4,4-^2^H_3_] 2-ketobutyrate (Marley et al. 2001). Perdeuterated, uniformly ^15^N, D-Glucose-1,2,3,4,5,6,6-d_7_ and selective γ_1_-^13^C Isoleucine Brd4-BD1 expression (concentrations in [g/l] used as above) was initialized by taking several colonies and inoculating them in a 10 mL M9-H_2_O minimal medium for 8 h at 37 °C shaking. From that culture, 250 µL were taken and inoculated in 10 mL fresh M9-D_2_O minimal medium, which was shaken overnight at 37 °C. At a cell density of ~ 2.5, the culture was taken and transferred to the final expression culture in a total 300 mL M9-D_2_O medium. The cells were grown until an OD_600nm_ of 0.7 and induced with 0.4 mM IPTG. Cells were harvested by centrifugation, lysed by sonication and the lysates were subsequently centrifuged. Proteins were purified from the supernatant by Ni^2+^ affinity chromatography. The purified protein was treated with TEV protease and again loaded onto a Ni^2+^ column to bind the cleaved His6-tag and the His6-tagged TEV protease. The flow-through containing Brd4-BD1 was concentrated and stored in 10 mM sodium phosphate buffer pH 7.5, 100 mM sodium chloride and 1 mM dithiothreitol (DTT). In the case of perdeuterated Brd4-BD1, after concentrating, the buffer was exchanged to D_2_O buffer (10 mM sodium phosphate buffer pH 7.5, 100 mM sodium chloride and 1 mM DTT). Purity was analyzed by SDS-Page. NMR samples of Brd4-BD1 were prepared in 10 mM sodium phosphate buffer containing 0.1–0.5 mM protein, 100 mM sodium chloride, 10% D_2_O, and 1 mM DTT. NMR samples of perdeuterated Brd4-BD1 were prepared in 10 mM sodium phosphate D_2_O buffer containing 0.2 mM protein, 100 mM sodium chloride, and 1 mM deuterated Tris(2-Carboxyethyl)phosphine:DCl-D16 (TCEP).

### NMR measurements

Carbon relaxation studies. All protein NMR measurements were conducted at 298 K on a Bruker Neo 600 MHz spectrometer equipped with a TXI RT probe head with perdeuterated and selective γ_1_-^13^C Isoleucine Brd4-BD1 sample concentration of 200 µM. For ^13^C relaxation studies, pulse sequence “hsqcctetgpsp” for the constant time ^1^H–^13^C HSQC with the constant time delays of 0.0133, 0.0266, 0.04 and 0.0532 s were used (Vuister and Bax 1992). For the relaxation measurement of ^13^C–^1^H_2_ heteronuclear triple/single quantum coherence, the pulse sequence “hmqcctetgp.2” (slightly modified) for the constant time ^1^H–^13^C HTQC was used (Marino et al. 1997). The constant time delays were set to 0.028, 0.042, 0.056, 0.07, 0.084, 0.098, 0.112, 0.128, 0.14, 0.154 s. To allow direct comparison of intensities, all spectra in a series were acquired with the same spectral parameters and the same settings for the receiver gain. Specifically, spectra were recorded using 106 (f1) × 1024 (f2) real points (CT-HSQC) and 144 (f1) × 1024 (f2) real points (CT-HTQC) with acquisition times of 0.06 × 0.01 s. 128 scans per fid were recorded with a recycle delay of 1 s. The pulse scheme of the CH_2_-TROSY experiment was applied as described by Miclet et al. (2004) and the corresponding spectrum recorded with a time delay of 0.028 s.

**Fig. 1:**
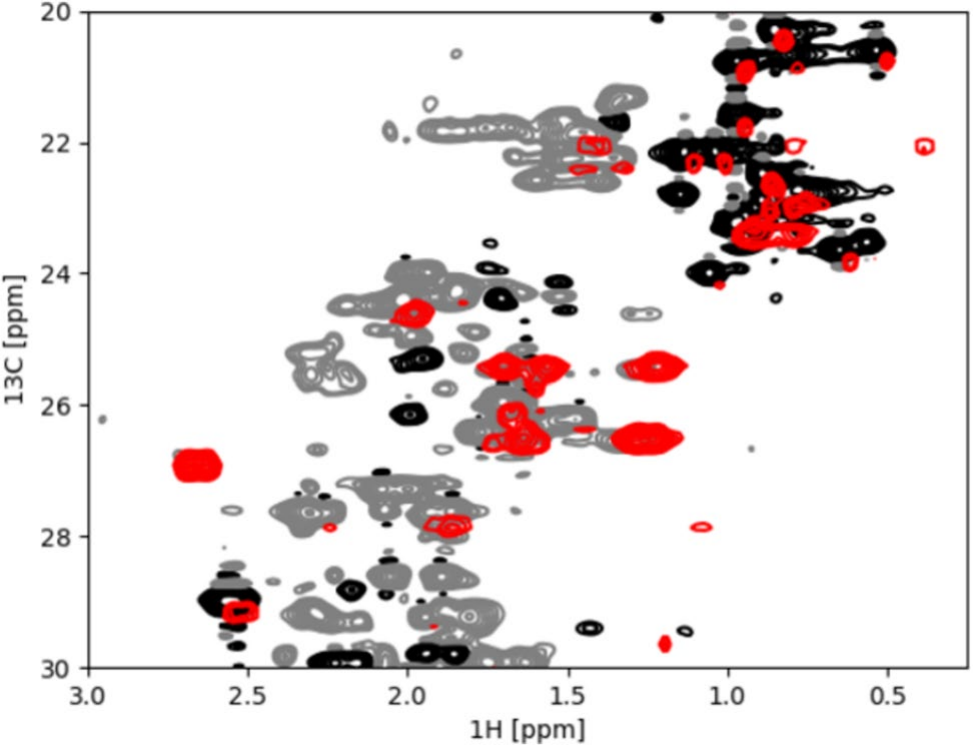
Overlay of ^1^H–^13^C-HSQC spectra of selectively Ile ^13^CH_2_ labeled Brd4-BD1 (red) onto the CT-^1^H–^13^C-HSQC of ^13^C-uniformly labeled Brd4-BD1 (^13^CH and ^13^CH_3_ signals in black and ^13^CH_2_ signals in grey), zoomed into the CH_2_ region

Protein-Ligand interaction studies. Protein-Ligand interaction measurements were conducted at 298 K on a Bruker Avance HD3 + 800 MHz spectrometer equipped with a TXI RT probe head with Brd4-BD1 sample concentration of 200 µM and ligand concentration of 1 mM. 2D ^1^H–^13^C HSQC spectra were acquired using the pulse sequence “hsqcetfpgpsi2” of the Bruker library (Palmer et al. 1992; Kay et al. 1992; Grzesiek and Bax 1993; Schleucher et al. 1994). Spectra were recorded using 128 (f1) × 1024 (f2) real points and acquisition times of 0.05 × 0.01 s. 32 scans were recorded per t1 increment with a recycle delay of 1 s.

### Analysis

NMR spectra were processed and analyzed with NMRPipe (Delaglio et al. 1995) and SPARKY (Goddard and Kneller 2006) and CCPNmr (Vranken et al. 2005). Unambiguous sequential assignment of the Isoleucine Cγ1 signals was performed with a series of three-dimensional NMR experiments (HNCACB, HN(CO)CACB, hCC-TOCSY-coNH and Hcc-TOCSY-coNH). ^13^C relaxation studies were performed by fitting the logarithmic intensities of the peaks to the linear regression model in RStudio (RStudio Team 2020) by taking the linearized logarithmic function as log[I(t)] = log(A) − t/T_2_.

## Results

To demonstrate the potential of our selectively labeled isoleucine precursor in protein dynamics studies, we incorporated [3-^13^C; 4,4,4-^2^H_3_] α-ketobutyric acid **8** into the bromodomain 1 of Bromodomain-containing protein 4 (Brd4-BD1).

Brd4-BD1 belongs to the family of bromodomain and extra-terminal domain (BET) proteins, which act as chromatin readers by binding to acetylated histones and therefore regulating gene expression (Hu et al. 2022). Brd4-BD1 contains seven Isoleucine residues, theoretically yielding 14 peaks. The incorporation of the ^13^C label into Brd4-BD1 was confirmed by a ^1^H–^13^C-HSQC spectrum (Fig. 1, in red). As expected, two cross peaks are obtained for the diastereotopic methylene protons of each Ile Cγ_1_ differing in the hydrogen dimension, but with the same carbon chemical shift. Note that the peaks of two of the Isoleucine Cγ_1_ groups are overlapping (presumably I100 and I101). However, spurious cross peaks are found in the methyl group region of the ^1^H–^13^C HSQC spectra (Fig. 1). In order to validate that these peaks are indeed natural abundance correlations of methyl CH_3_ obtained from the expression with D-Glucose (^12^C) in H_2_O medium (SI Fig. 1, in black), we incorporated the [3-^13^C; 4,4,4-^2^H_3_] α-ketobutyric acid also in D_2_O minimal media (SI Fig. 1, in red). As shown in SI Fig. 1 in red, the natural abundance peaks are suppressed by deuteration, and additionally, no metabolic scrambling of the precursor can be observed.

**Fig. 2:**
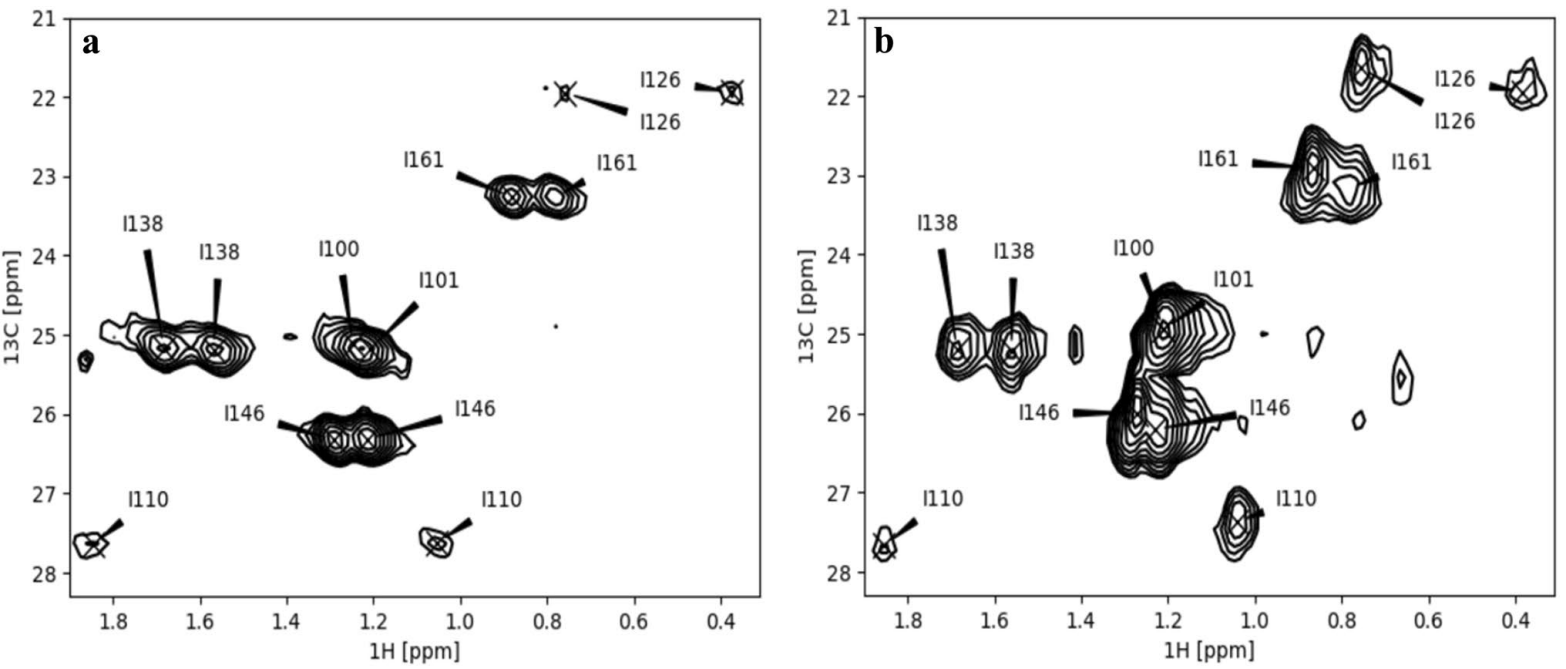
Comparison between CT-HTQC (ct delay 0.028 s) (**a**) and CT-HSQC (ct delay 0.013 s) (**b**) spectra of perdeuterated ^15^N-Brd4-BD1-Ile-^13^C*γ*1

**Fig. 3:**
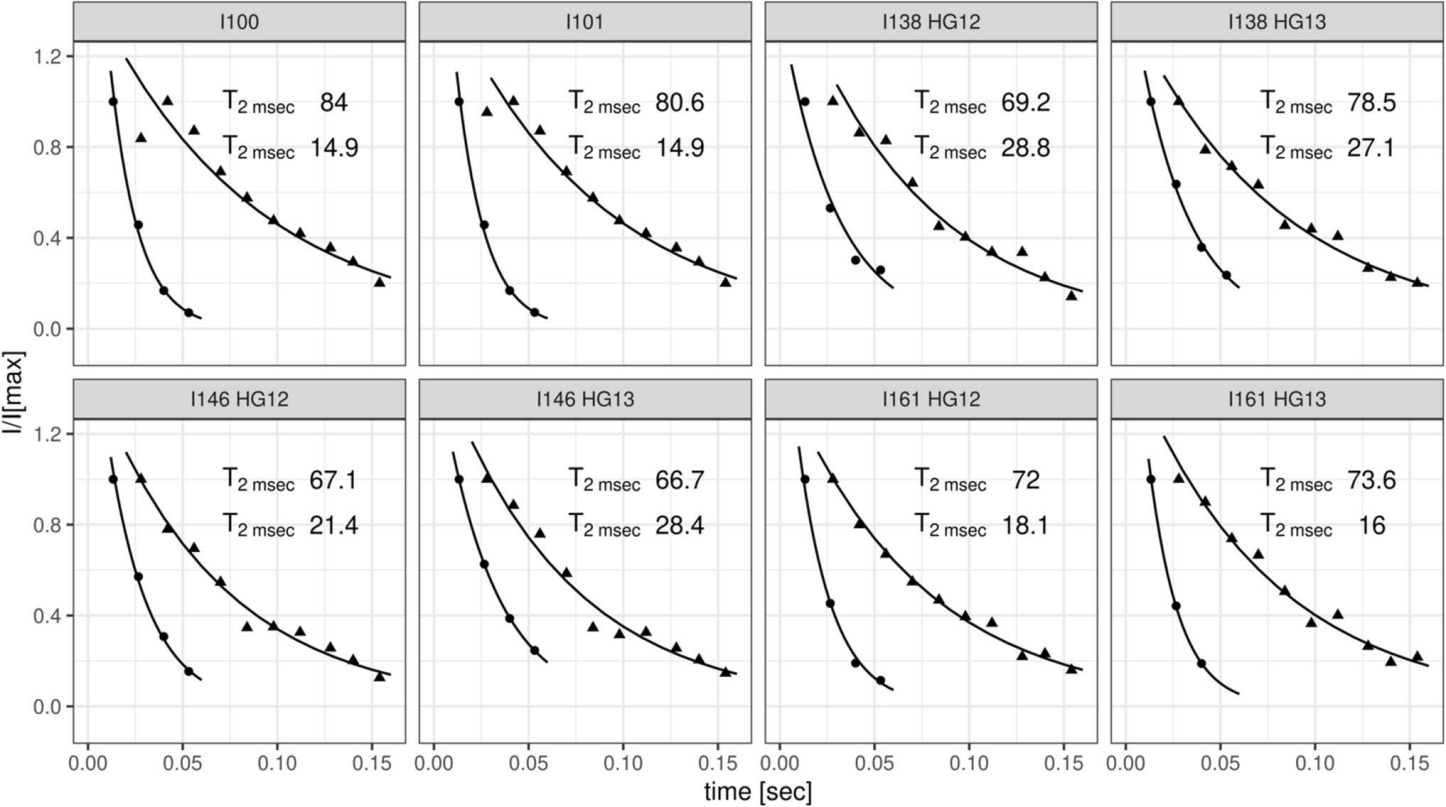
Extracted ^13^C relaxation times for specific Ile residues in perdeuterated Brd4-BD1. Dots represent relaxation times extracted from CT-HSQC, whereas pyramids represent rates extracted from CT-HTQCs

The availability of ^13^CH_2_ labeled Ile-residues offers the possibility to create heteronuclear multiple-quantum (triple/single-quantum, T(S)QC) coherences in a straightforward manner. It is well-known that triple/single quantum ^13^CH_2_ coherences relax more slowly than the corresponding single quantum coherences of the individual ^13^C and ^1^H spins (Grzesiek and Bax 1995; Grzesiek et al. 1995; Marino et al. 1997; Ruschak et al. 2010; Tugarinov and Kay 2013). Effective relaxation rates were measured by recording a series of ^1^H–^13^C-CT-HSQCs and ^1^H–^13^C-CT-HTQCs spectra (Fig. 2) with different relaxation delays (see experimental section). Figure 3 shows a comparison of the rates extracted from the ^1^H–^13^C-CT-HSQC and ^1^H–^13^C-CT-HTQC, respectively. It can clearly be seen that the ^13^C relaxation times for heteronuclear T/SQC extracted from the ^1^H–^13^C-CT-HTQC were increased on average by a factor of three compared to the ^13^C-T_2_ from the ^1^H–^13^C-CT-HSQC, as expected from theory (Grzesiek and Bax 1995; Grzesiek et al. 1995; Marino et al. 1997; Ruschak et al. 2010; Tugarinov and Kay 2013). It should be noted that data for residues I110 and I126 could not be analyzed as their signals are barely above the noise level. Interestingly, these two residues are also buried inside the hydrophobic core of Brd4-BD1, whereas the other five Isoleucine residues are found rather on the surface or at the flexible parts of the protein, thus suggesting the existence of considerable exchange broadening due to conformational averaging.

Selectively Ile-labeled and otherwise highly deuterated samples are moreover particularly interesting for applying specific transverse-relaxation-optimized NMR spectroscopy (TROSY) methods, which have been introduced to improve the sensitivity and resolution of methylene NMR studies (Miclet et al. 2004). We recorded a corresponding CH_2_-TROSY spectrum of the Ile ^13^CH_2_ Brd-BD1 sample expressed in D_2_O/deuterated glucose, which shows additional gain in spectral resolution – even relative to the HTQC spectrum (especially in the ^1^H dimension by virtually eliminating the large geminal ^2^J_HH_) as a result of the exclusive TROSY selection of the slowest relaxing multiplet component. Upon comparing the signal-to-noise ratios of the different experiments, it becomes evident that HTQC performs better in this regard compared to both CH_2_-TROSY and HSQC (SI Fig. 2).

**Fig. 4:**
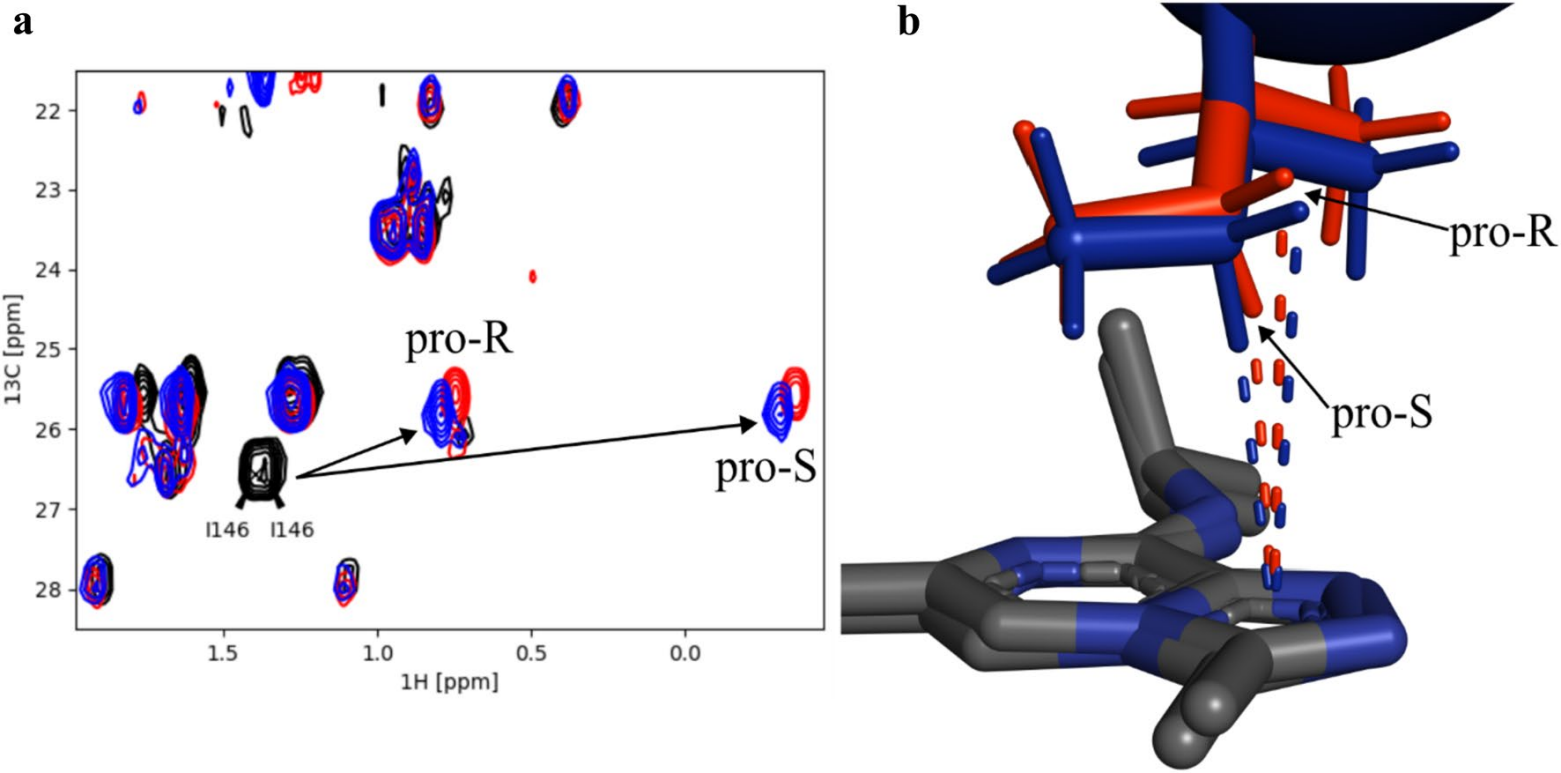
**a** ^1^H-^13^C-HSQC overlay of Brd4-BD1-Ile-^13^Cγ1 (black) with Ligand **A** (red) and Ligand **B** (blue). **b** Zoom into the Brd4-BD1 binding pocket with an overlay of Ligand **A** (red, 6XV3) and **B** (blue, 6XUZ). Dashed lines indicate the distances between the ligand ring center and the two diastereotopic protons of Ile146. The prochiral designation of the methylene group is indicated in both figures

 Finally, in order to test whether the methylene group of Isoleucine can be used as a reporter of CH-π interactions, we decided to measure ^1^H–^13^C-HSQCs with ligands **A** and **B**, which are nanomolar Brd4-BD1 binders (Platzer et al. 2020). Figure 4a shows the chemical shift changes of ^15^N-Brd4-BD1-Ile-^13^Cγ1 with 1 mM ligand **A** (red) and **B** (blue). The ligand induced proton chemical shift perturbation (CSP) of the Ile146 methylene resonances result in 1.71 ppm for ligand **A** and 1.78 ppm for ligand **B** for one hydrogen atom and 0.59 ppm and 0.66 ppm for the other hydrogen, respectively. These significant CSPs point towards an ideally oriented CH-π interaction between the Ile146-C_γ1_H_2_ and the ligand aromatic system, as favorable CH-π stacking orientations are correlated with high upfield shifts. Taking a closer look at the crystal structure of Brd4-BD1 in complex with both ligands (Fig. 4b), the hydrogen of the methylene group (pro-S) is directly positioned over the centroid of the triazole ring, whereas the pro-R hydrogen atom is further away from the ligand. This information enables us therefore to also stereo-specifically assign the hydrogens of the isoleucine methylene groups. In order to correlate the observed CSPs of the Ile146 methylene resonance with the relative orientation of the ligand in the binding pocket of Brd4-BD1, we used the Pople equation (Pople 1956; Platzer et al. 2020). The geometric parameters were taken from the published X-ray structures (see SI Table 2), resulting in a calculated CSP for the pro-S hydrogen of Ile146 of 1.1 ppm and 1.5 ppm for ligand **A** and **B**. For the hydrogen atom in pro-R configuration, the calculated CSPs are 0.5 ppm and 0.6 ppm, respectively. This finding shows that the experimentally derived CSPs match well with the calculated, again supporting the presence of a beneficial CH-π stacking orientation.

## Discussion and conclusion

The novel α-ketobutyric isotopologue reported here provides an economic tool to implement methylene labeling in isoleucine side chains. This precursor is directly applicable to *E.coli*-based expression systems, as shown by incorporation into the human protein Brd4-BD1, allowing observation of seven distinct Isoleucine Cγ1–H_2_ pairs in an otherwise crowded ^1^H–^13^C-HSQC spectra (Fig. 1). The incorporation of the precursor into Brd4-BD1 by expression in D_2_O minimal media results in simplified protein spectra against a deuterated background with favourable ^13^C relaxation properties and enhanced signal-to-noise ratio. Our experiments further exemplify the benefit of the deuterated Isoleucine Cγ1 labeled Brd4-BD1 by studying its carbon transverse relaxation properties by measuring ^13^C single and ^13^C/^1^H heteronuclear multiple quantum coherences. Comparison of the extracted effective ^13^C relaxation times shows an increase by a factor of ~ 3.5 going from ^13^C SQC to heteronuclear triple quantum coherences (TQC). The larger ^13^C relaxation times in the TQC experiment might allow for interesting applications such as paramagnetic relaxation enhancement (PRE), residual dipolar couplings (RDC) and conformational exchange via CPMG-type measurements. In practice, this has to be balanced against other experimental factors such as potentially higher overall intrinsic signal intensity in the HSQC spectra (due to signal improvement by Rance/Kay type gradient schemes) but holds the promise of application to large molecular weight systems. Most importantly, however, the large upfield chemical shift observed for the pro-S methylene proton in the BRD4-ligand complex convincingly demonstrates the usefulness of the Isoleucine methylene group as an excellent reporter for CH-π interactions.

It can thus be anticipated that particularly protein-ligand binding studies will considerably benefit from the availability of the new Isoleucine precursor. The facile and efficient introduction of this novel precursor to realize new Isoleucine isotopologues in a target protein represents a valuable and general tool to fine-tune NMR studies and decipher protein dynamics, allosteric mechanisms, and binding interactions.

### Electronic supplementary material

Below is the link to the electronic supplementary material.

Supplementary Material 1
