# Supplementary material for: Synthesis of a 13C-methylene-labeled isoleucine precursor as a useful tool for studying protein side-chain interactions and dynamics
Source: J Biomol NMR. 2023 Oct 11;78(1):1–8. doi: 10.1007/s10858-023-00427-2 (PMC10981609; doi:10.1007/s10858-023-00427-2)
Supplement: Supplementary file 1 — Supplementary Material 1 [file 10858_2023_427_MOESM1_ESM.docx]

**Synthesis of a ^13^C-Methylene-Labeled Isoleucine Precursor as a Useful Tool for Studying Protein Side-chain Interactions and Dynamics**

Theresa Höfurthner^1,3^, Giorgia Toscano^2,3^, Georg Kontaxis^4^, Andreas Beier^1^, Moriz Mayer^5^, Leonhard Geist^5^, Darryl B. McConnell^5^, Harald Weinstabl^5^, Robert Konrat^1,4*^, Roman Lichtenecker^2*^

*^1^ Christian Doppler Laboratory for High-Content Structural Biology and Biotechnology, Department of Structural and Computational Biology, Max Perutz Laboratories, University of Vienna, Campus Vienna Biocenter 5, 1030 Vienna (Austria)*

*^2^ Christian Doppler Laboratory for High-Content Structural Biology and Biotechnology, Institute of Organic Chemistry, University of Vienna, Währingerstraße 38, 1090 Vienna (Austria)*

*^3^ Vienna Doctoral School in Chemistry (DoSChem), University of Vienna, Währingerstraße 42, 1090 Vienna (Austria)*

*^4^ Max Perutz Laboratories, Department of Structural and Computational Biology, Campus Vienna Biocenter 5, 1030 Vienna*

*^5^ Boehringer Ingelheim RCV GmbH & Co. KG, Dr. Boehringer Gasse 5-11, 1121 Vienna*

** corresponding authors Dr.Roman Lichtenecker,* [*roman.lichtenecker@univie.ac.at*](mailto:roman.lichtenecker@univie.ac.at)*; Univ.Prof. Dr. Robert Konrat, robert.konrat@univie.ac.at*

**General Information – Organic Synthesis**

Unless otherwise stated, all reagents and reactants were purchased from commercial suppliers and used without further purification. All solvents were distilled before use. Iodomethane-^13^C and Iodomethane-d_3_ were purchased by Merck Isotec®. Oxygen- and moisture sensitive reactions were carried out under an argon atmosphere and yields refer to pure compounds. The reactions were monitored via thin layer chromatography (TLC) on silica gel 60 with fluorescent indicator UV254 by MACHEREY-NAGEL GmbH & Co. KG. Visualization of the compounds was carried out using an UV-lamp (254 nm) and by application of specific reagents: H_3_PMo_12_O_40_ 10 % in ethanol with subsequent heating using a heat-gun. Flash column chromatography was performed on silica gel 60 (0.040-0.063 mm) from Merck. Freeze-drying was performed by cooling in liquid nitrogen and subsequent application of high vacuum (oil pump). ^1^H and ^13^C 1D NMR spectroscopic data were recorded on a Bruker AVANCE-DRX 400 MHz spectrometer. NMR solvent signals were calibrated to 7.27 ppm (CDCl_3_), 4.79 ppm (D_2_O). Chemical shifts (δ) are given in ppm (s = singlet, d = doublet, dd = doublet of doublets, m = multiplet) and reported relative to the residual solvent peaks. Coupling constants (J) are given in Hertz (Hz). High resolution mass spectrometry experiments were performed using electrospray ionization (ESI, 3 keV, in the positive or negative ion mode) or electron ionization (EI, 70 eV).

**Abbreviations:** PPh_3_ …Triphenylphosphine, EtOAc …Ethyl acetate, Et_2_O …Diethyl ether, DCM …Dichloromethane, RT …room temperature, THF …Tetrahydrofuran, NaHMDS …Sodium bis(trimethylsilyl)amide, sat …saturated

**Synthetic Procedures**

**Synthesis of Compound 2**

The procedure was adapted from literature (Werkhoven et al. 1999). A solution of 12,3 g (1,05 eq) of PPh_3_ in 25 ml of EtOAc, 8,23 g (44,6 mmol) of tert-butyl-2- bromoacetate 1 were added and the solution was stirred at room temperature overnight. The next day, the white precipitate was filtered off and washed three times with Et_2_O. After drying, the precipitate was dissolved in DCM and 100 ml of 1 M (2,2 eq) NaOH solution was added. The mixture was stirred vigorously for 15 min, and the two phases separated. The aqueous phase was extracted twice with DCM. All organic phases were combined and dried over MgSO_4_. Evaporating the solvents in vacuum yielded a yellow solid, which was taken up in a minimum amount of DCM. Precipitation of the product was promoted by adding a layer of heptane and stirring for one hour at 0 °C. The precipitate was filtered and washed with heptane to yield *tert*-butyl 2-(triphenylphosphoranylidene)acetate **2** as a white powder. Yield: 16.78 g (quantitative) ^1^H NMR (CDCl_3_, 400 MHz) δ 7,95 (3H, m), 7.77 – 7.49 (12H, m), 5.67 (1H, m), 1.21 & 0.98 (9H, two broad s).

**Synthesis of Compound 3**

The experimental procedure was conducted as reported in the literature (Werkhoven et al. 1999). A solution of 2,9 g (7,7 mmol) (*tert*-butoxycarbonylmethylene)triphenylphosphorane **2** in 15 ml DCM, 1 g (0,9 eq) ^13^CH_3_I was added. After stirring the solution overnight, the solvent was removed under reduced pressure. The yellow residue was taken up in 30 ml of DCM and shaken vigorously with a solution of 560 mg (2 eq) NaOH in 10 ml of H_2_O in a separation funnel. The aqueous phase was extracted 2x with DCM. The combined organic phases were dried over MgSO_4_ to afford a clear, yellow product solution, which was used for the next step without any further purification.

**Synthesis of Compound 4**

A constant stream of O_3_ from an ozone generator was purged through the solution of the ylid **3** in DCM (resulting from the preceding reaction) at -78 °C until the solution turned blue. Subsequently, a stream of argon was then purged through the solution until the blue colour vanished. 2,22 mg (1,2 eq) of PPh_3_ were added and the solution was stirred under inert conditions for another 15 min at -78 °C. The mixture was then brought to RT and stirring was continued for two hours. The bulk solvent was carefully removed under reduced pressure. [3-^13^C] *Tert*-butyl pyruvate **4** was obtained by bulb-to-bulb distillation (100 mbar, up to 90 °C) as a colorless liquid. Yield: 782.3 mg (70 % over 2 steps) ^1^H NMR (CDCl_3_, 400 MHz) δ 2.42 (3H, d, 1J_CH_ = 129.2 Hz), 1.56 (9H, s). ^13^C (CDCl_3_, 100 MHz) δ 84.13, 26.36, 27.93.

**Synthesis of Compound 5**

Compound 5 was prepared similar as reported in (Hajduk et al. 2000). To a solution of 311 mg (2,14 mmol) [3-^13^C] *tert*-butyl pyruvate **4** in 5 ml Et_2_O, 200 µl (1,2 eq) N,N-dimethylhydrazine were added under argon atmosphere. The mixture was stirred at RT overnight. The next day, the solution was diluted with Et_2_O and then washed 2x with small amounts of H_2_O. The aqueous phase was extracted 3x with small amounts of Et_2_O. The organic phases were pooled, dried over MgSO_4_, and concentrated under reduced pressure. [3-^13^C] *Tert*-butyl 2-(2,2-dimethylhydrazono)propanoate **5** was purified by bulb-to-bulb distillation (10 mbar, up to 110 °C) and obtained as a yellow liquid. Yield: 303 mg (76 %) of two isomers in an approx. ratio of 5:1. ^1^H NMR (CDCl_3_, 400 MHz) Isomer 1: δ 2.79 (6H, s), 2.06 (3H, d, 1J_CH_ = 129.7 Hz), 1.53 (9H, s); Isomer 2: 2.51 (1.2H, s), 2.04 (0.6H, d, 1J_CH_ = 129.7 Hz), 1.53 (1.8H, s). ^13^C NMR (CDCl_3_, 100 MHz) δ 15.95, 19.48, 26.61, 28.09, 47.06, 47.52, 81.64, 82.61. HRMS δ (M + Na)+ for C_8_^13^CH_18_N_2_O_2_ calculated 210.1294; found 210.1298.

**Synthesis of Compound 6**

A solution of 303 mg (1,62 mmol) of hydrazone **5** in 15 ml anhydrous THF was set under argon atmosphere and cooled to -78 °C. 3,25 ml (2 eq) of 1 M NaHMDS in THF/hexanes were added over the course of 30 seconds. Stirring was continued for 2 min and 102 µl (1 eq) CD_3_I were quickly added. After 5 min of additional stirring, the reaction was quenched with 5 ml of H_2_O and brought to RT. After phase separation, the aqueous phase was extracted 4x with Et_2_O. The combined organic phases were washed with H_2_O and dried over MgSO_4_. The solvent and silyl side products were removed under reduced pressure (50 mbar, 40 °C) for several hours until mass consistency was reached. Bulb-to-bulb distillation (10 mbar, up to 110 °C) afforded [3-^13^C; 4,4,4-^2^H_3_] *Tert*-butyl 2-(2,2-dimethylhydrazono)butanoate **6** as a clear, yellow liquid. Yield: 264.65 mg of two isomers in a ratio of approx. 4:1 (80 %) ^1^H NMR (CDCl_3_, 400 MHz) Isomer 1: δ 2.77 (6H, s), 2.53 (2H, d, 1J_CH_ = 129.7 Hz), 1.52 (9H, s); Isomer 2: 2.50 (1.2H, s), 2.35 (0.5H, d, 1J_CH_ = 129.7 Hz), 1.52 (2.2H, s); ^13^C NMR (CDCl_3_, 100 MHz) δ 82.59, 81.71, 47.89, 32.40, 28.45, 26.97, 22.12, 19.93, 18.52, 16.13, 10.28; HRMS (M + Na)+ for C_9_^13^CH_17_D_3_N_2_O_2_+Na calculated 227.1633; found 227.1638.

**Synthesis of Compound 7**

The synthesis of α-ketoester 7 was performed as described in the literature (Lichtenecker et al. 2004). A solution of 190 mg (0,93 mmol) of hydrazone **6** in 25 ml Et_2_O was shaken vigorously with 1,12 ml of 1 M HCl in a separation funnel for 1 minute. The phases were separated, and the aqueous phase was extracted 2x with Et_2_O. The combined organic phases were washed 1x with sat. NaHCO_3_ solution and 1x with brine. The solution was then dried over MgSO_4_ and bulk solvent was carefully removed under reduced pressure. The product was further purified by column chromatography (silica gel, 10 % diethyl ether in pentane), which afforded 230 mg of a clear, colourless solution of [3-^13^C; 4,4,4-^2^H_3_] *tert*-butyl-2-ketobutanoate **7**. Yield: 143 mg (95 %) calculated from ^1^H-NMR spectrum. ^1^H NMR (CDCl_3_, 400 MHz) δ 2.78 (2H, d, 1J_CH_ = 127.0 Hz), 1.54 (9H, s) HRMS (M + Na)+ for C_7_^13^CH_11_D_3_O_3_ calculated 185.1062; found 185.1054.

**Synthesis of Compound 8**

The α-ketoacid 8 was prepared as previously described (Lichtenecker et al. 2004). A solution of 143 mg (0,70 mmol) **7** in 20 ml of a 50:50 mixture of Et_2_O and DCM was cooled to 0 °C. HCl gas (generated from slowly dropping concentrated HCl into concentrated sulphuric acid) was bubbled through this solution for 15 minutes at a moderate rate. Then, the reaction vessel was sealed and stirred at RT for 1 h. The solution was then cooled again to 0 °C, and the process was repeated 3 times, while stirring was continued overnight after the last cycle. The solvent was removed under reduced pressure to afford [3-^13^C; 4,4,4-^2^H_3_] 2-ketobutyric acid as a clear, slightly yellowish viscous liquid. Yield: 66.8 mg (90 %) ^1^H NMR (CDCl_3_, 400 MHz) δ 3.00 (2H, d, 1J_CH_ = 127.6 Hz). HRMS (M-H)- for C_3_^13^CH_3_D_3_O_3_ calculated 105.0466, found 105.0459.

**Synthesis of Compound 9**

The α-keto-acid **8** was suspended in 5 ml of H_2_O and neutralized with 0.1 M NaOH carefully to a pH of 7. The sodium salt was lyophilised to afford [3-^13^C; 4,4,4-^2^H_3_] sodium 2-ketobutyrate **9** as a white, powdered solid.

Yield: 72.5 mg (90 %). ^1^H NMR (D_2_O, 400 MHz) δ 2.73 (2H, d, 1J_CH_ = 127.1 Hz).

**NMR Spectra:**


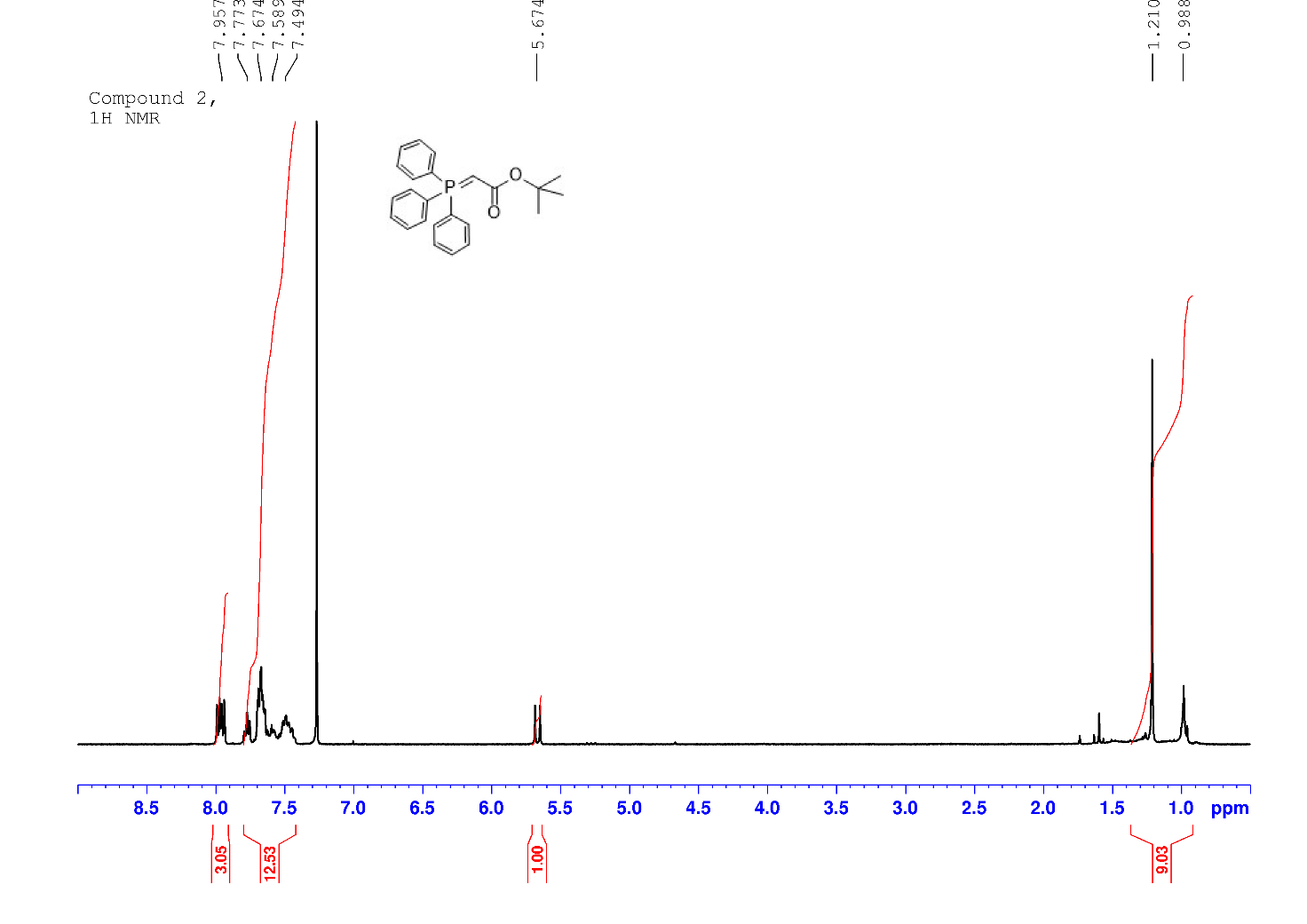


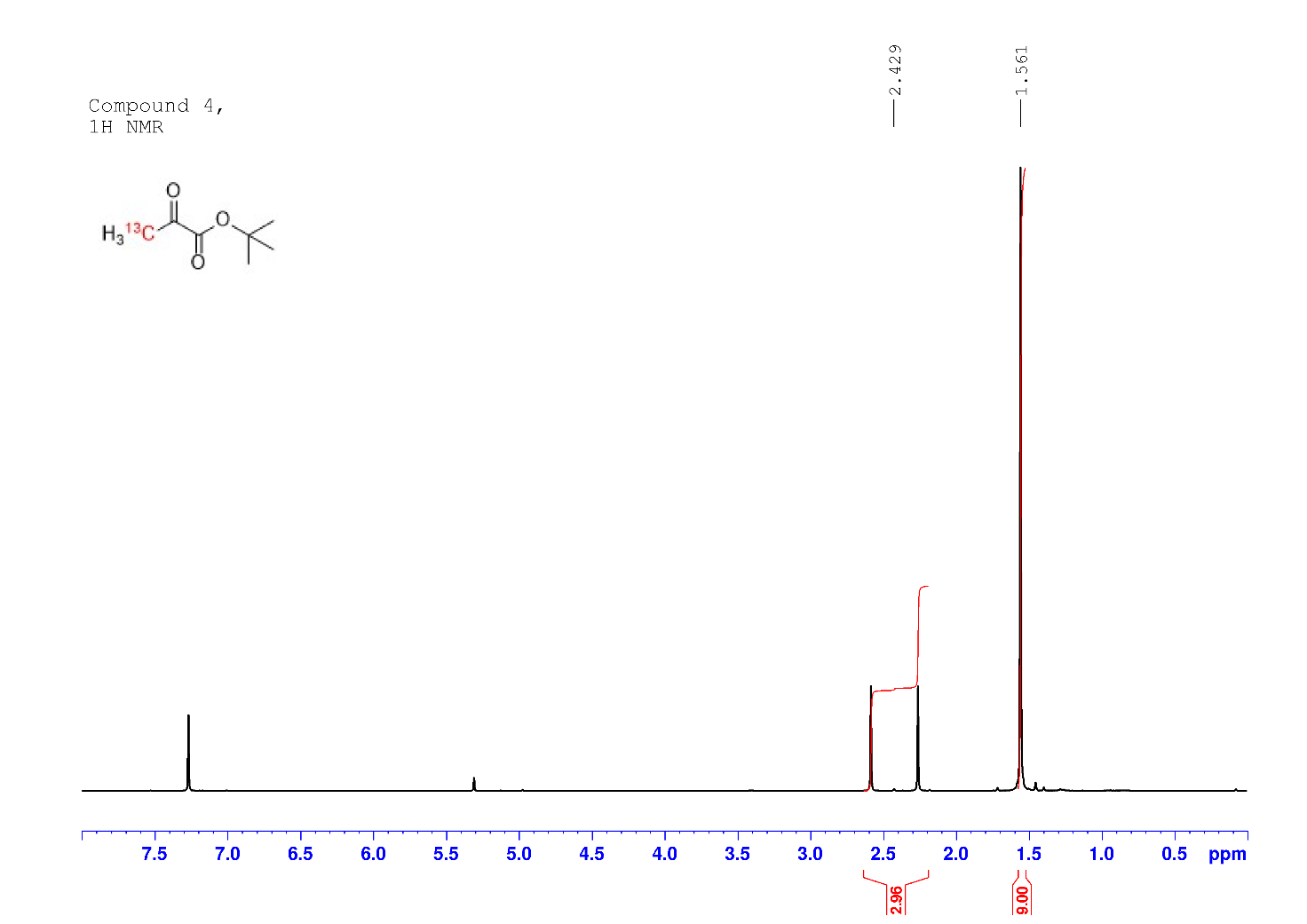


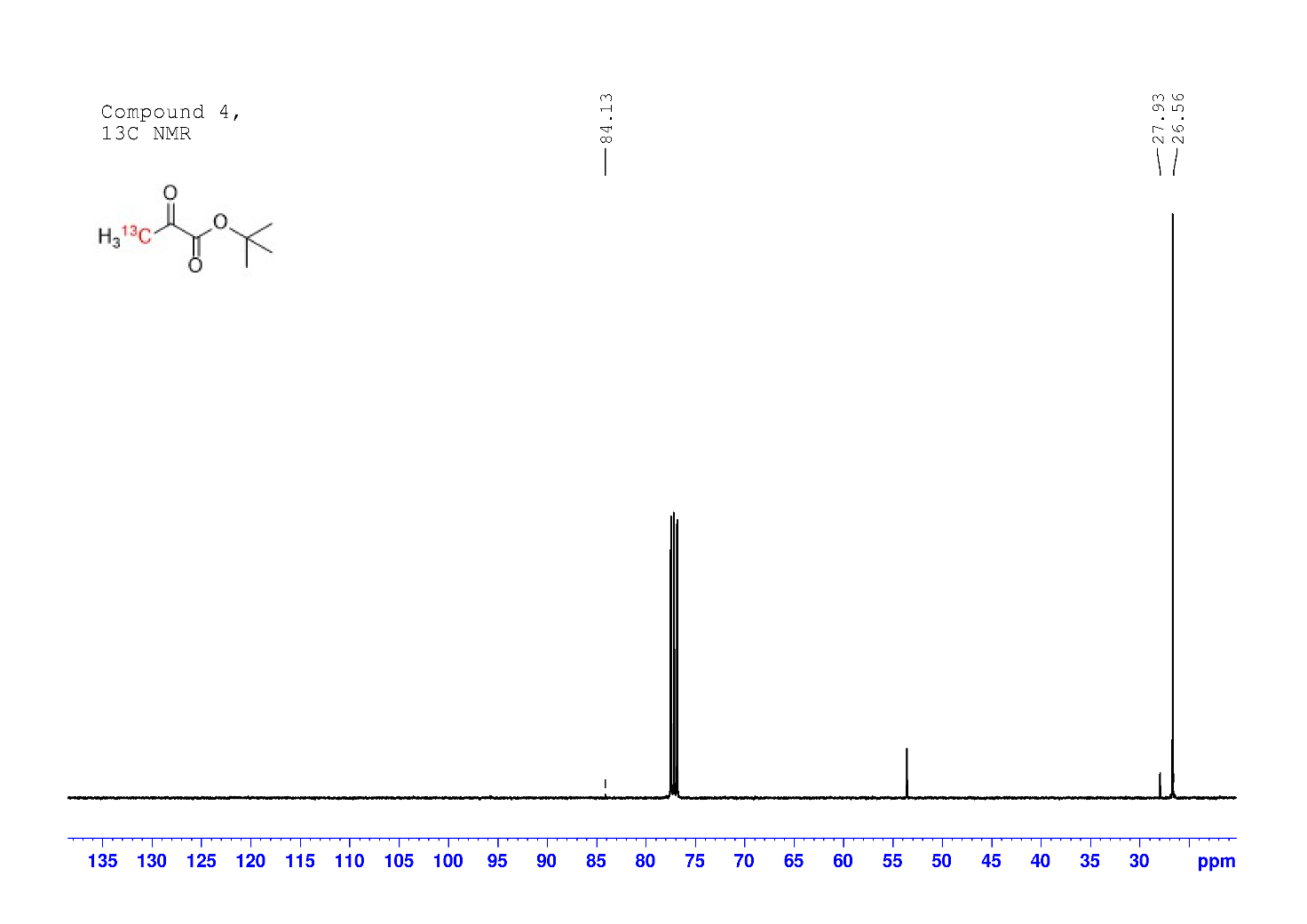


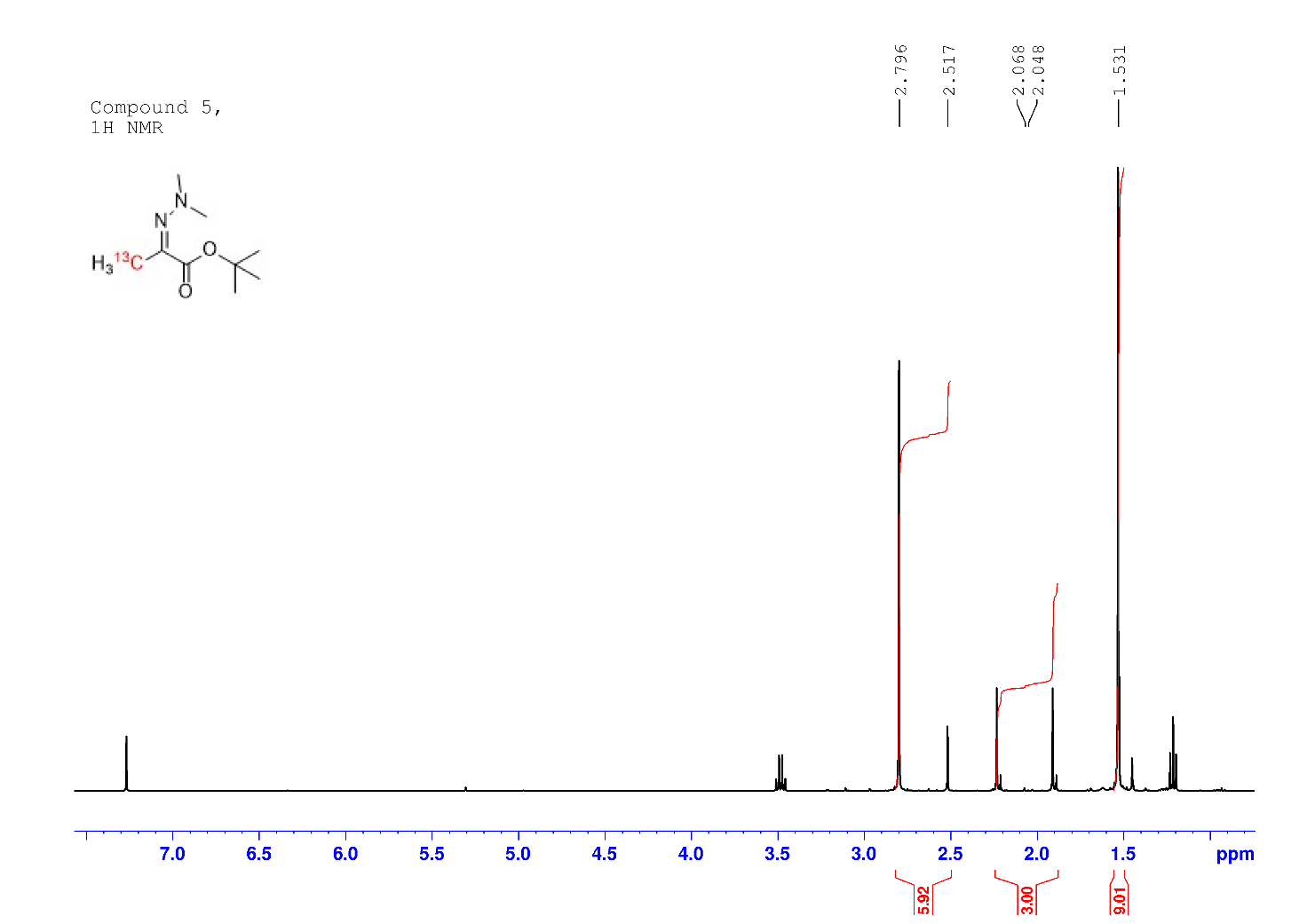


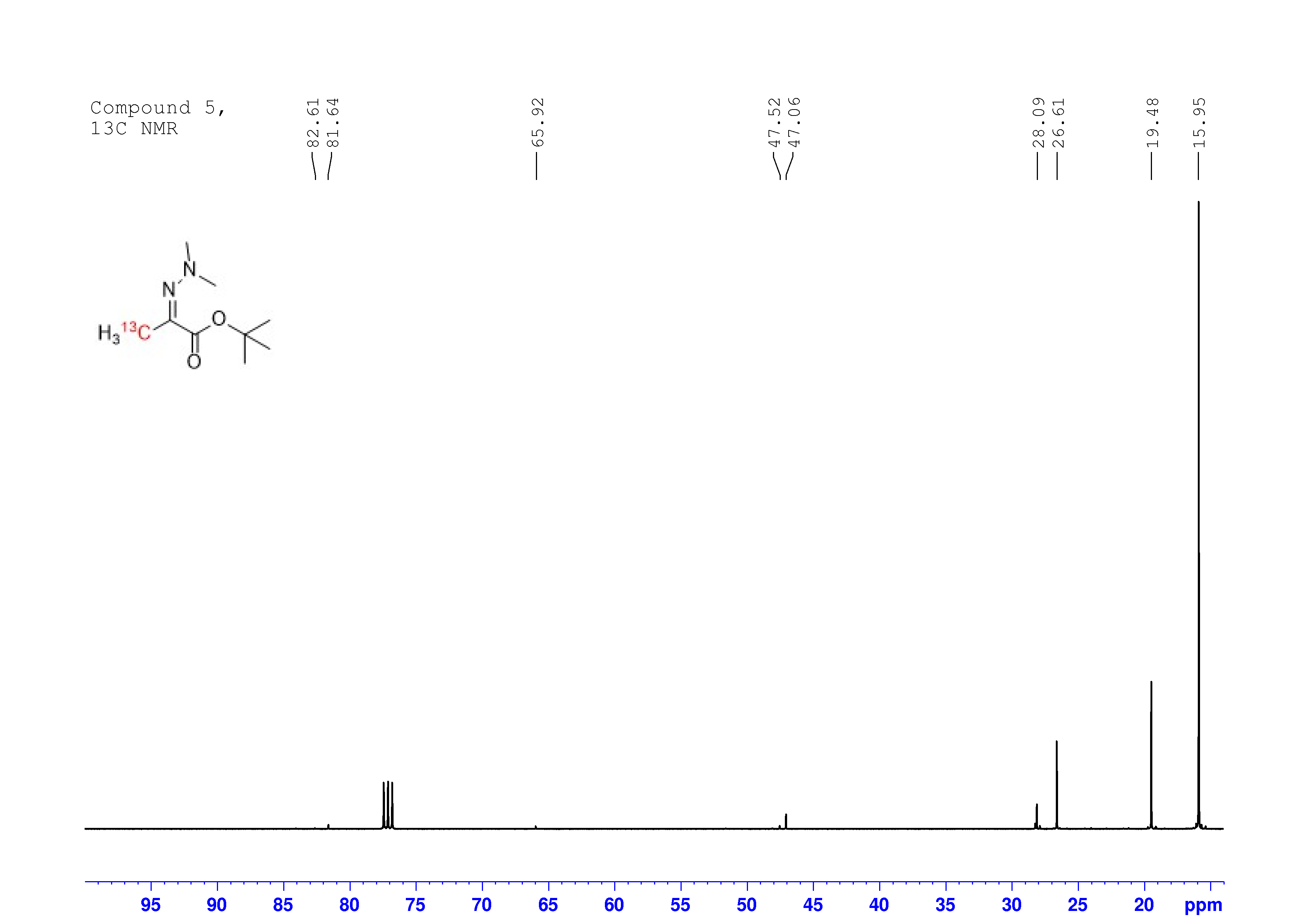


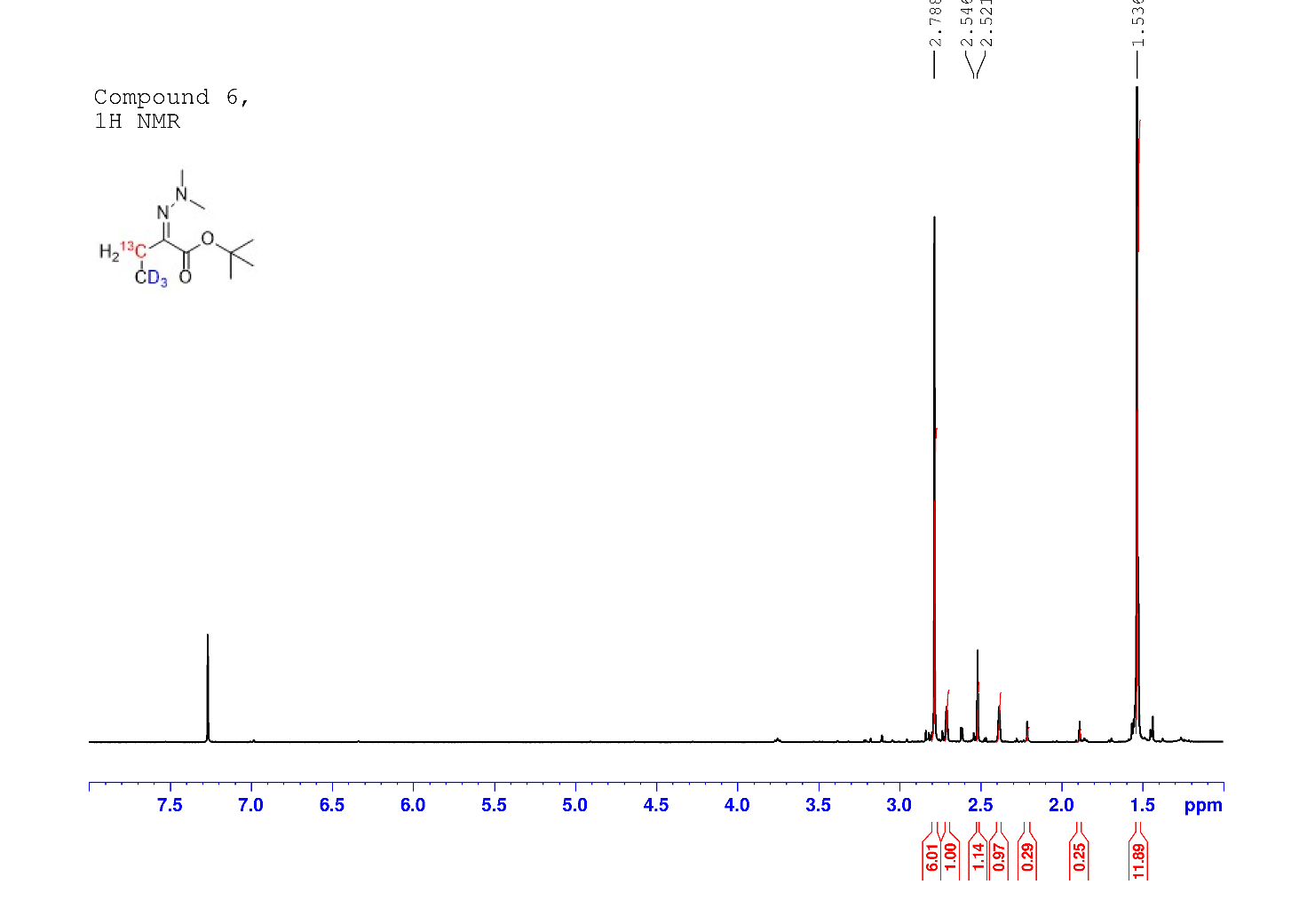


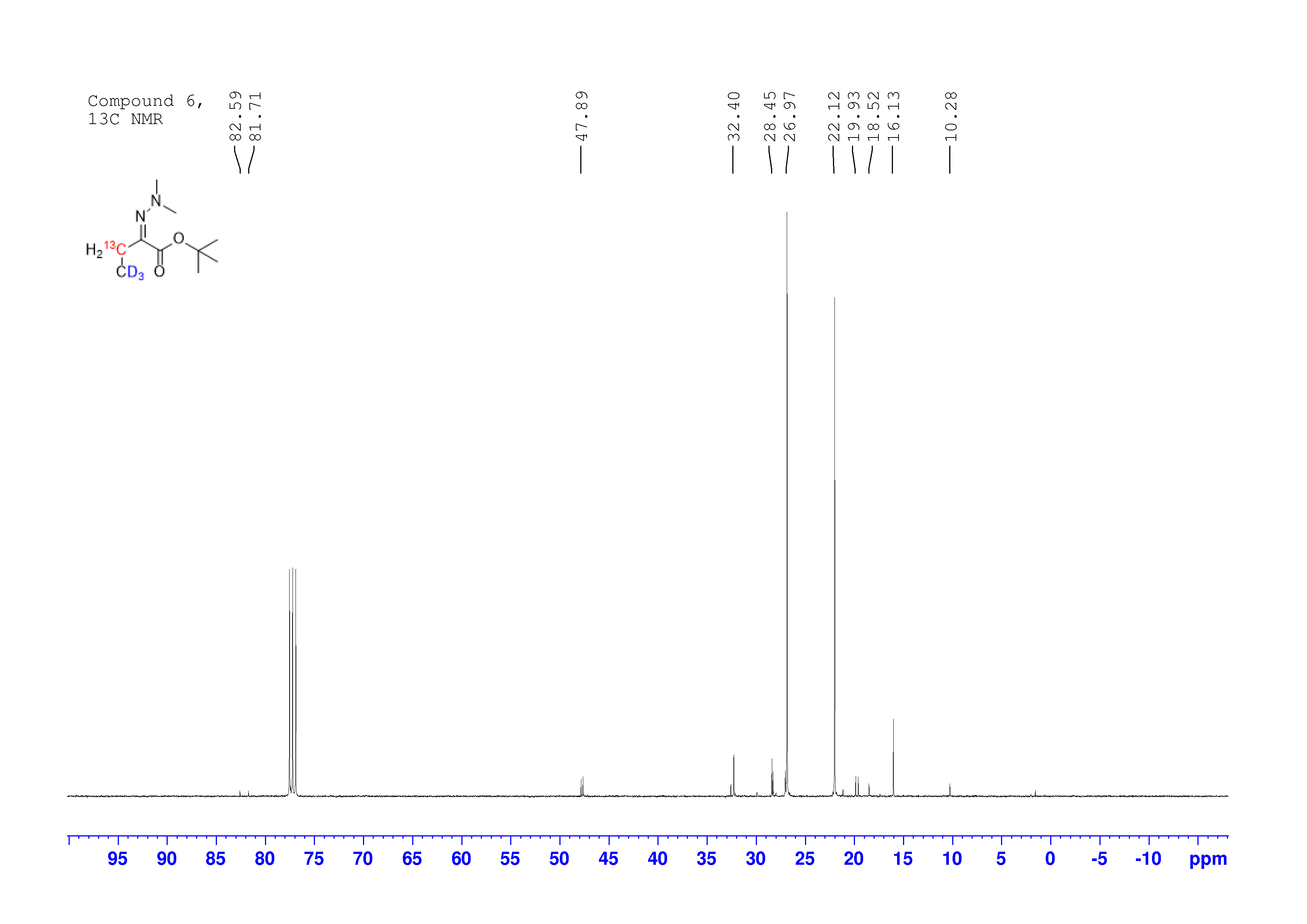


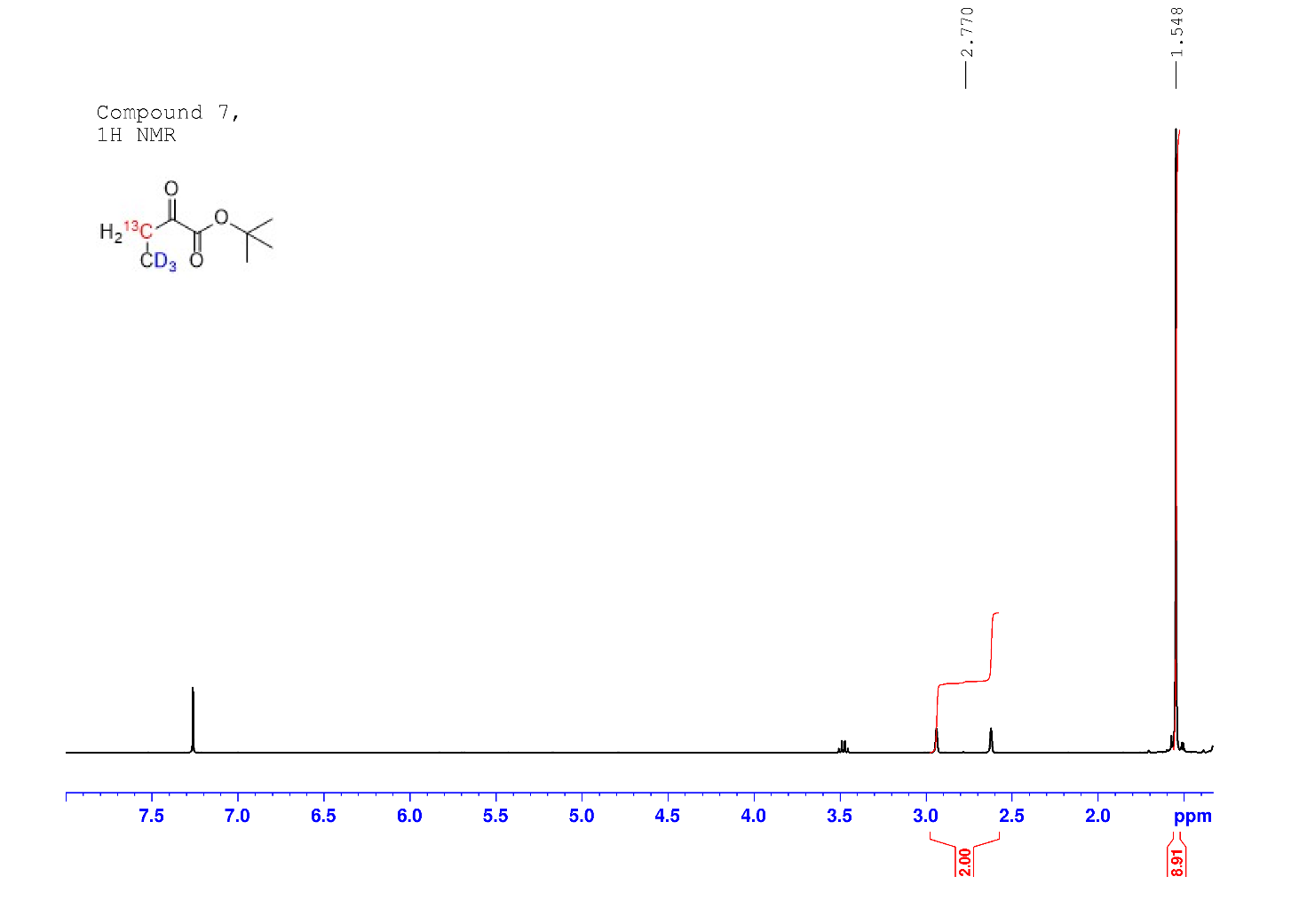


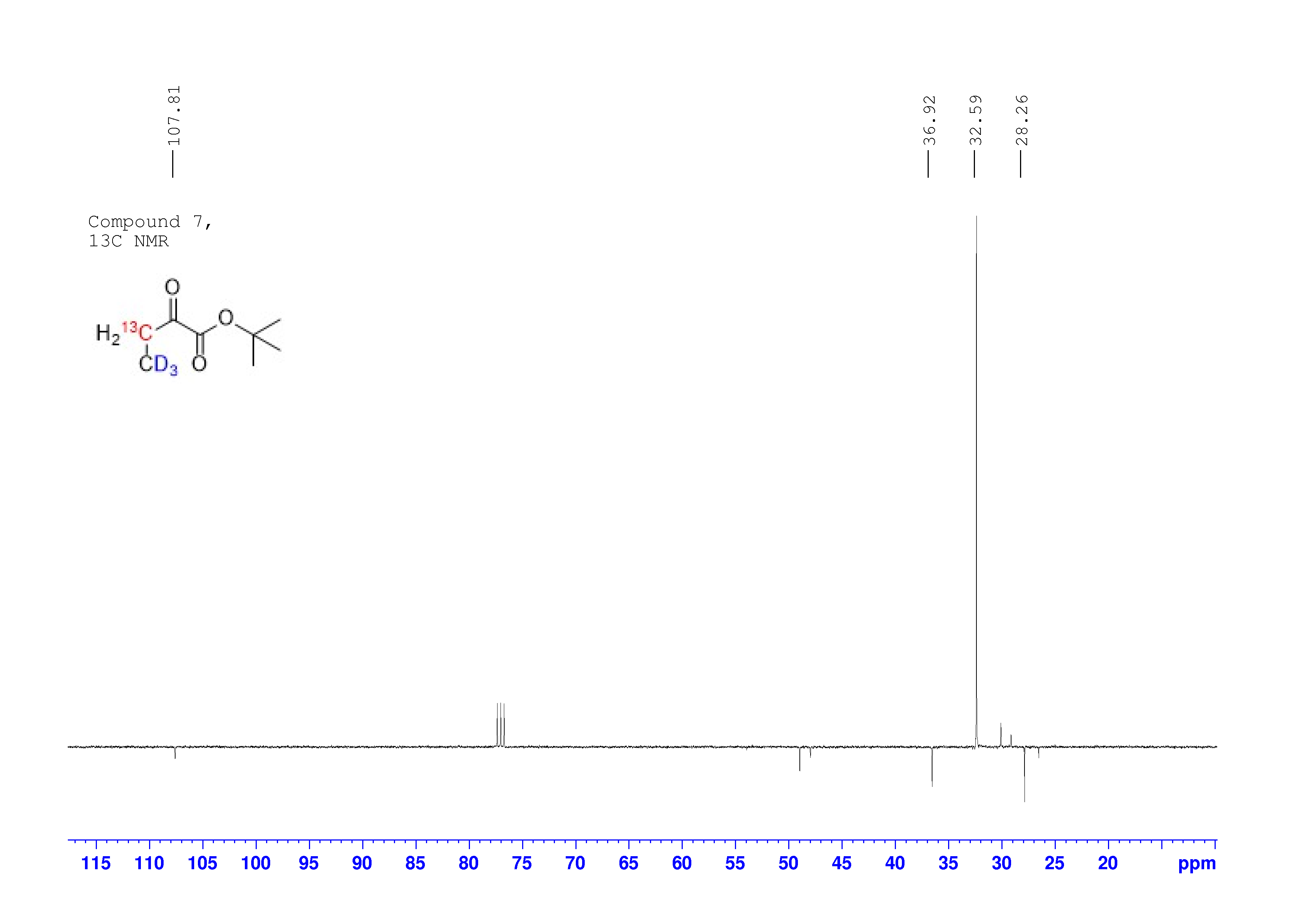


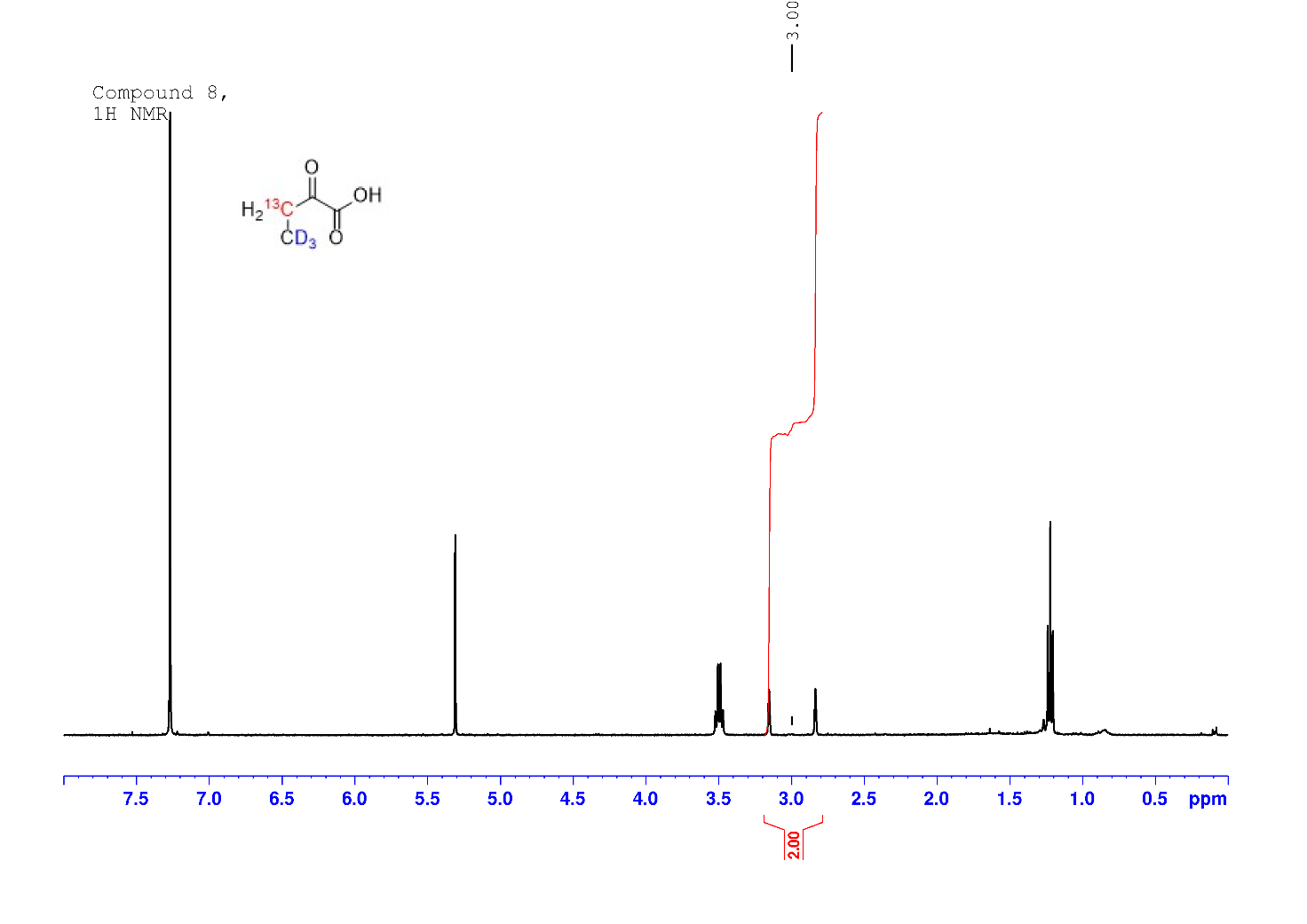


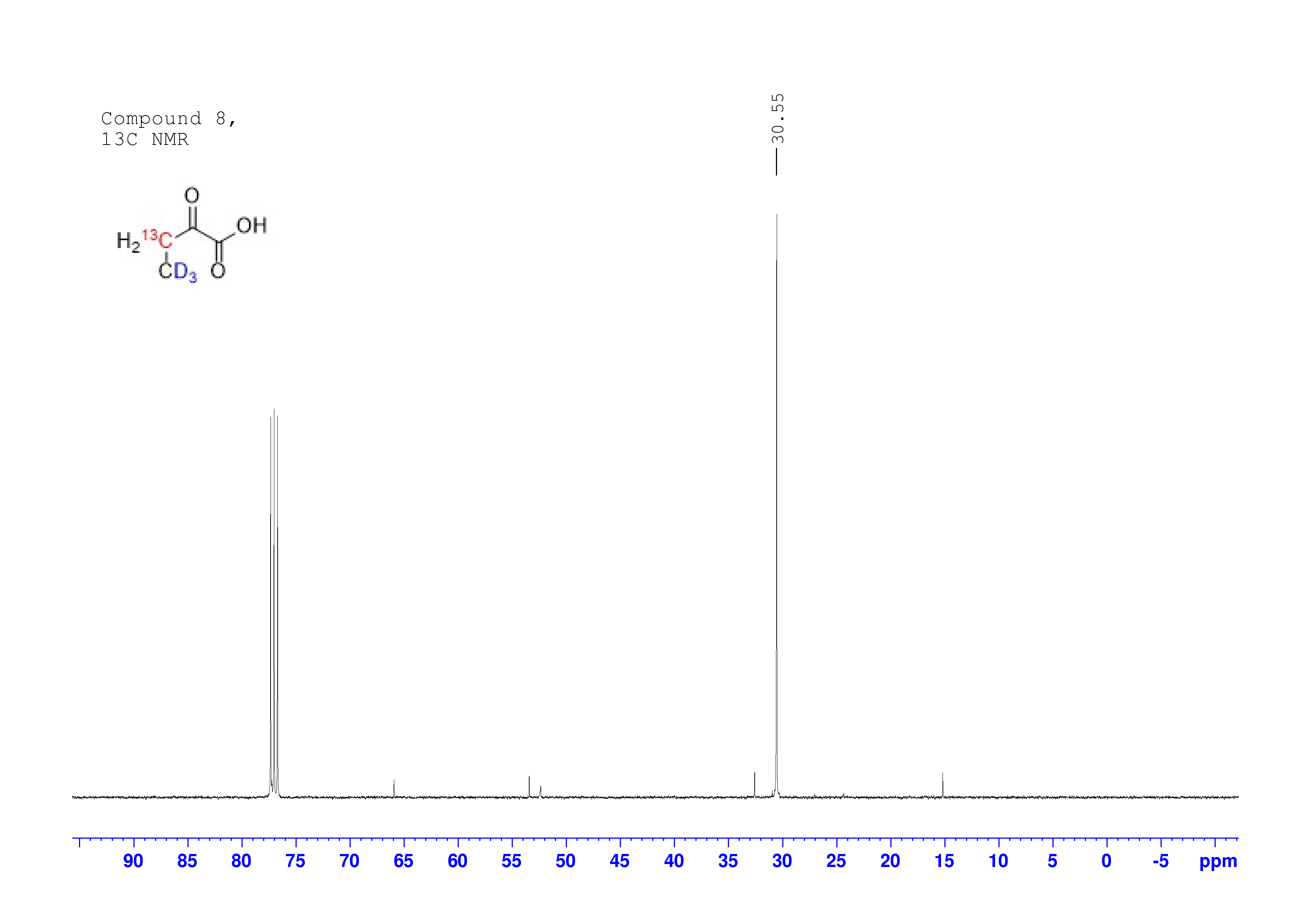


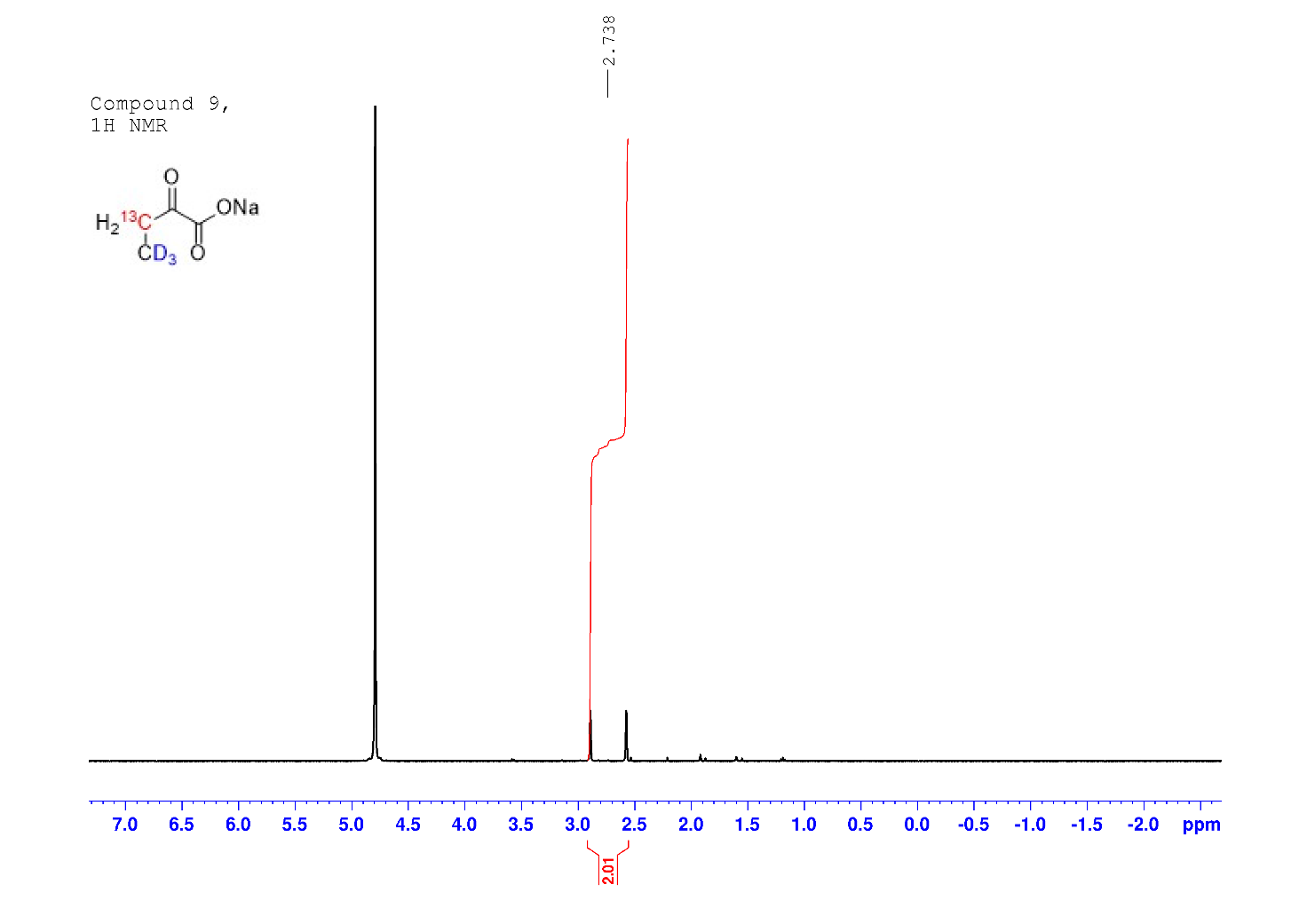


**Incorporation of [3-^13^C; 4,4,4-^2^H_3_] α-ketobutyric acid**

As mentioned in the main text, spurious cross peaks are found in the methyl group region of the ^1^H-^13^C HSQC spectra. In order to verify that these peaks are indeed natural abundance correlations of methyl CH_3_ obtained from the expression with D-Glucose (^12^C) in H_2_O medium (Figure 1, in red), we incorporated the [3-^13^C; 4,4,4-^2^H_3_] α-ketobutyric acid also in D_2_O minimal media. As shown in SI Figure 1 in red, the natural abundance peaks are suppressed by deuteration, and additionally, no metabolic scrambling of the precursor can be observed.


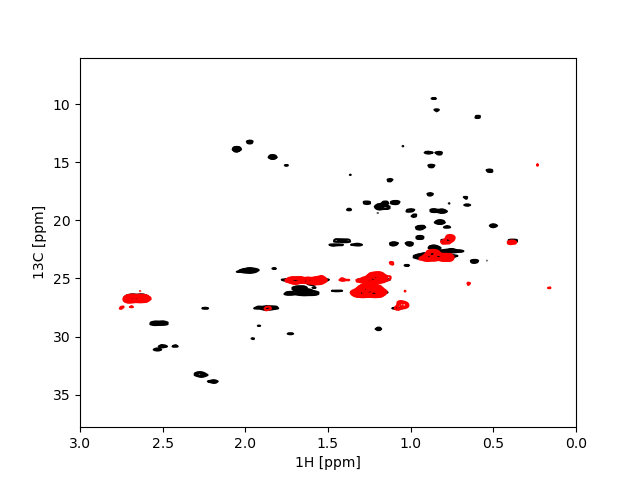


SI Figure 1 Overlay of ^1^H-^13^C-HSQC spectra of selectively labeled Brd4-BD1, expressed in H_2_O (black) and selectively labeled Brd4-BD1, expressed in D_2_O (red)

**^13^C Transverse Relaxation Studies**

Analysis of the ^13^C relaxation studies were performed by fitting the logarithmic intensities of the peaks (SI Figure 1) to the linear regression model in RStudio (RStudio Team 2020). The linearized logarithmic function was taken as log[I(t)]=log(A)-t/T_2._ The peak intensities were normed to the maximum intensity of the same peak in each time step. The extracted T_2_ values are displayed in milliseconds, as well as the residual standard error and the multiple R-squared of the fit (arbitrary unit).

|  | HTQC | | | HSQC | | |
| --- | --- | --- | --- | --- | --- | --- |
| Residue | T_2_ [ms] | S | R^2^ | T_2_ [ms] | S | R^2^ |
| ILE101 | 80.6 | 0.10 | 0.969 | 14.9 | 0.06 | 0.998 |
| ILE126 HG12 | 144.3 | 0.13 | 0.850 | 16.2 | 0.21 | 0.967 |
| ILE138 HG13 | 78.5 | 0.08 | 0.980 | 27.1 | 0.04 | 0.997 |
| ILE146 HG13 | 66.7 | 0.13 | 0.965 | 28.4 | 0.01 | 0.999 |
| ILE161 HG13 | 73.6 | 0.11 | 0.966 | 16.0 | 0.01 | 0.999 |
| ILE161 HG12 | 72 | 0.09 | 0.981 | 18.1 | 0.12 | 0.990 |
| ILE100 | 84.0 | 0.13 | 0.945 | 14.9 | 0.06 | 0.998 |
| ILE146 HG12 | 67.1 | 0.13 | 0.965 | 21.4 | 0.05 | 0.998 |
| ILE138 HG12 | 69.2 | 0.14 | 0.955 | 28.8 | 0.18 | 0.945 |
| ILE126 HG13 | 83.2 | 0.23 | 0.753 | -6.4 | NaN | 1 |
| ILE110 HG13 | 113.4 | 0.2 | 0.726 | 14.3 | NaN | 1 |
| ILE110 HG12 | 84.6 | 0.13 | 0.919 | 11.7 | NaN | 1 |

SI Table 1: extracted T_2_ relaxation times for each peak observed in the CT-HSQC and CT-HTQC.


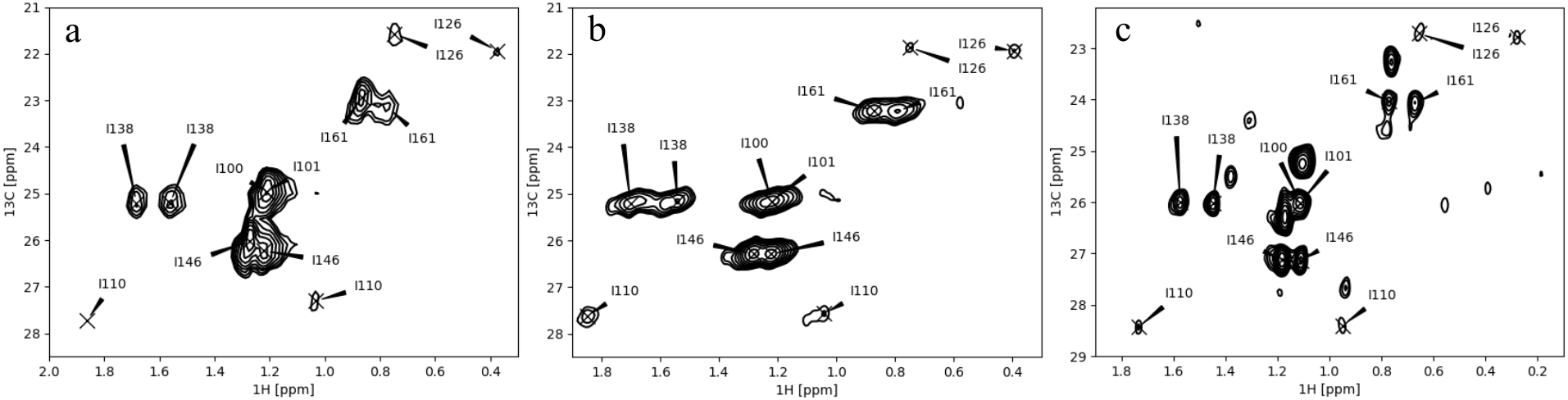


SI Figure 2 Comparison of ^1^H-^13^C-CT-HSQC (a), ^1^H-^13^C-CT-HTQC (b) and CH_2_-CT-TROSY (c) spectra of perdeuterated and selectively labeled Brd4-BD1 at a constant time delay of 0.028s sec for each spectra. Signals in (c) are shifted compared to (a) and (b) due to J_CH_ coupling. Residual peaks in the CH_2_-TROSY (c) are presumably due to incomplete suppression because of J-mismatch (in the S3E filter), imperfections in the TROSY element and differential ^1^H relaxation times.

**Protein-Ligand Interaction** (Pople 1956; Platzer et al. 2020)

For detailed description of the equation, please refer to (Pople 1956; Platzer et al. 2020).

| **Ligand** | **Structure** | **CSP [ppm]**  **pro-R** | **H—X [Å]**  **pro-R** | **H—Y [Å]**  **pro-R** | **θ [rad]**  **pro-R** | **Δσ [ppm]**  **pro-R** |
| --- | --- | --- | --- | --- | --- | --- |
|  |  | **pro-S** | **pro-S** | **pro-S** | **pro-S** | **pro-S** |
| **A** |  | 0.66 | 0.41 | 4.17 | 0.09 | 0.50 |
|  |  | 1.78 | 0.97 | 2.91 | 0.32 | 1.11 |
| **B** |  | 0.59 | 0.28 | 3.93 | 0.07 | 0.61 |
|  |  | 1.72 | 0.96 | 2.55 | 0.36 | 1.50 |

SI Table 2 Structures, chemical shifts and geometrical parameters used in this study

**References**

Hajduk PJ, Augeri DJ, Mack J, et al (2000) NMR-Based Screening of Proteins Containing 13 C-Labeled Methyl Groups. J Am Chem Soc 122:7898–7904. https://doi.org/10.1021/ja000350l

Lichtenecker R, Ludwiczek ML, Schmid W, Konrat R (2004) Simplification of Protein NOESY Spectra Using Bioorganic Precursor Synthesis and NMR Spectral Editing. J Am Chem Soc 126:5348–5349. https://doi.org/10.1021/ja049679n

Platzer G, Mayer M, Beier A, et al (2020) PI by NMR: Probing CH–π Interactions in Protein–Ligand Complexes by NMR Spectroscopy. Angew Chemie Int Ed 59:14861–14868. https://doi.org/10.1002/anie.202003732

Pople JA (1956) Proton Magnetic Resonance of Hydrocarbons. J Chem Phys 24:1111–1111

RStudio Team (2020) RStudio: Integrated Development for R

Werkhoven TM, van Nispen R, Lugtenburg J (1999) Specific Isotope Enrichment of Methyl Methacrylate. European J Org Chem 1999:2909–2914. https://doi.org/10.1002/(sici)1099-0690(199911)1999:11<2909::aid-ejoc2909>3.3.co;2-x
